# Peripheral infusion of human umbilical cord mesenchymal stem cells rescues acute liver failure lethality in monkeys

**DOI:** 10.1186/s13287-019-1184-2

**Published:** 2019-03-12

**Authors:** Gang Guo, Xiang Zhuang, Qing Xu, Zhenru Wu, Yongjie Zhu, Yongjie Zhou, Yuanmin Li, Yanrong Lu, Bo Zhang, Prue Talbot, Jiayu Liao, Junjun She, Hong Bu, Yujun Shi

**Affiliations:** 1 Laboratory of Pathology, Key Laboratory of Transplant Engineering and Immunology, NHC, West China Hospital, Sichuan University, 37 Guoxue Road, Chengdu, 610041 China; 2 Department of Pathology, West China Hospital, Sichuan University, Chengdu, 610041 China; 3 Sichuan Stem Cell Bank & Sichuan Neo-Life Stem Cell Biotech Inc., Chengdu, 610037 China; 4 Department of Cell Biology & Neuroscience, University of California, Riverside, CA 92521 USA; 5 The UCR Stem Cell Center and Core of University of California, Riverside, CA 92521 USA; 6 Department of Bioengineering, University of California, Riverside, CA 92521 USA; 7 Department of Talent Highland, First Affiliated Hospital of Xi’an Jiao Tong University, Xian, 710061 China; 8 Department of General Surgery, First Affiliated Hospital of Xi’an Jiao Tong University, Xian, 710061 China

**Keywords:** Acute liver failure, Non-human primate, Interleukin-6, Monocyte, Mesenchymal stem cell

## Abstract

**Background:**

Acute liver failure (ALF) is a complicated clinical syndrome associated with high mortality, with liver transplantation as the only treatment option. Treatment of mesenchymal stem cells has shown a potential therapeutic option for acute liver failure. However, the lack of random clinical trials and large non-human primate studies makes it necessary to assess the efficacy and safety in the clinic.

**Methods:**

We treated the monkeys with peripheral delivery of human umbilical MSCs (hUC-MSCs) and investigated the role of hUC-MSCs in modulating the progress of acute liver failure.

**Results:**

The use of early peripheral infusion of human umbilical cord MSC infusion did not improve liver regeneration or modulate adaptive immunity. However, it significantly suppressed the hepatic aggregation and maturation of circulating monocytes and their IL-6 secretion, greatly improving liver histology, systemic homeostasis, and survival.

**Conclusions:**

Our study reveals the critical role of monocyte-derived IL-6 in initiating and accelerating acute liver failure and hUC-MSC treatment can disrupt the development of the inflammatory cascade by inhibiting monocyte activation. Early hUC-MSC treatment disrupts the development of the inflammatory cascade, indicating a potential clinical solution for acute liver failure.

**Electronic supplementary material:**

The online version of this article (10.1186/s13287-019-1184-2) contains supplementary material, which is available to authorized users.

## Background

Acute liver failure (ALF), caused by sudden and severe hepatic injury, is a complicated clinical syndrome composed of metabolic and immunological dysfunction, hepatic encephalopathy, coagulopathy, sepsis, and multi-organ failure. It causes over 60% mortality in western countries if liver transplantation is not provided. However, the shortage of donor livers and life-long immunosuppression have restricted the use of this intervention. The alternative strategies for liver transplantation, such as hepatocyte transplantation, bio-artificial liver support systems, and tissue-engineering livers, have been shown as a potential treatment option for ALF [1, 2]. Nonetheless, the efficacy and safety of these treatment options need to undergo many preclinical studies before applying in clinical stage. Thus, it is an urgent requirement to develop some new therapeutic methods for this fatal syndrome.

The pathophysiological process underlying ALF is still far from clear [2–4]. Given massive parenchymal necrosis and hepatic decompensation are the principle cause of ALF, however, immune system disarrangement, particularly the excessive activation of the innate immune system and the systemic release of inflammatory factors, which is described as a “cytokine storm” [5, 6], has been regarded as the core event in the process. The resident hepatic macrophages, known as Kupffer cells, play essential role in the production of various factors that initiate and exacerbate the uncontrolled hypercytokinemia [7, 8].

We previously established a non-human primate (*Macaca mulatta*) model of ALF, in which we induced ALF with a single intraperitoneal injection of low-dose α-amatoxin and lipopolysaccharide (LPS) [9, 10]. Following the injection of toxins, the monkeys displayed changes in clinical features, hepatic indexes, histopathology, imaging, and life span that are typical features of the progress observed in the clinical ALF [9]. Moreover, monkey is a species with metabolic and physiological properties similar to those of humans, indicating that our animal model is appropriate for exploring the pathophysiology of ALF and for evaluating potential therapeutic strategies. Based on this model, we have recently reported that among the dozens of increased inflammatory factors, IL-6 (interleukin 6) is the most immediately and dramatically increased cytokine before the appearance of typical changes in serum indices and liver histology, which acts as a critical trigger that flames the cytokine storm. Most interestingly, we observed that circulating monocytes (c-Mos), rather than Kupffer cells, serve as the principal producer of IL-6 before their hepatic aggregation and mature differentiation [9]. Therefore, c-Mos might act as a potential therapeutic target for this lethal syndrome, and our findings also encourage early therapies that against the activation of c-Mos prior to the full development of a cytokine storm.

Mesenchymal stem cells are a type of somatic stem cells derived from mesodermal tissues, including bone marrow, fat, cord blood, umbilical cord, placenta, etc. MSC-based therapies have been widely used for many diseases [11–13]. Moreover, numerous registered clinical trials centered on MSC-based treatments are currently underway worldwide. The therapeutic effects of MSCs have been extensively evaluated for acute liver injury by using animal models and in several clinical pilot studies [10, 14–16]. Interestingly, the results from these studies have demonstrated that MSCs can protect liver injury and promote liver repair, differentiate into hepatocytes, and suppress inflammatory reactions [12, 17, 18]. Thus, MSCs may represent a potential therapeutic option for treating ALF. However, it is necessary to accumulate the efficacy and safety of MSC-based therapies for ALF preclinically before testing in clinical stage.

It has been reported that Wharton’s jelly is one of the main sources of extracting human MSCs [19]. In recent years, considerable interest has been given to explore the therapeutic effects of stem cells due to the exciting properties. Approximately, one cord provides 100 million primary human umbilical cord MSCs (hUC-MSCs), with extensive in vitro proliferation capacity and after 5-passage culture, they can grow up to 1–10 billion cells [20]. Previous studies showed that hUC-MSCs can differentiate into adipocytes, osteocytes, chondrocytes, neurons, and oligodendrocytes. Compared with MSCs derived from other mesodermal tissues, hUC-MSCs possess a higher degree of stemness and provoke lower levels of immunogenicity [21, 22]. In addition, hUC-MSCs can secrete a large number of cytokines and have no tumorigenic effects [19, 20]. These characteristics, along with the ease by which hUC-MSCs are isolated from a tissue with unlimited availability, have generated much enthusiasm regarding their potential applications in cell-based therapies. This expectation is being supported by both preclinical and clinical data [23].

Here, using a toxin-induced monkey model of ALF, we explored the efficacy and safety of hUC-MSC-based therapy for ALF. Our work demonstrates that peripheral infusions of hUC-MSCs profoundly suppressed the activation of c-Mos and their IL-6 secretion, which resulted in the prevention of the development of lethal ALF in monkeys. These results, in combination with those demonstrating the efficacy and safety approach, indicate that hUC-MSC-based therapies are promising evidence which need further investigations and validation before being applied in clinical practice.

## Materials and methods

### Monkeys

The animal protocols used in this study were approved by the Institutional Animal Care and Use Committee of the Traditional Chinese Medicine National Center (Chengdu, China) (Protocol: IACUC-2012001C). Adult healthy experimental rhesus monkeys were provided by Chengdu Ping’an Experimental Animal Reproduction Center (license no.: SCXK (CHUAN) 2014-013, Chengdu, China). For a detailed information of the monkeys and their treatments, refer to Supplemental materials and methods Additional file 1: Table S1. To establish a toxin-induced ALF monkey model, the α-amatoxin (25 μg/kg bodyweight, Alexis Biochemicals, Lausen, Switzerland) and LPS (1 μg/kg bodyweight, Sigma-Aldrich, St. Louis, MO) were diluted in 50 ml of physiological saline and slowly infused into the peritoneal cavity, which is the same as our published article [9].

### hUC-MSCs (human umbilical cord mesenchymal stem cells)

Human umbilical cords were donated by women who underwent cesarean sections. Informed consent was obtained from the subjects’ families. hUC-MSCs were collected at the Sichuan Stem Cell Bank, Chengdu, China and cultured with serum-free medium (Stem cell 05420MesenCultTM-XF Medium, StemRD). For hUC-MSC quality control, cultured cells at the third passage (P3) were tested to determine the expression levels of surface markers by flow cytometry (Additional file 1: Figure S1A). To determine the multi-potential capacity, hUC-MSCs were cultured in the appropriate conditional medium to induce osteogenic and adipogenic differentiation (Additional file 1: Figure S1B). Moreover, viral factors, pathogenic organisms, and endotoxin levels were monitored (Additional file 1: Table S2). hUC-MSCs were used at P3–5 only when their viability was higher than 95% in all subsequent experiments.

### Delivery of hUC-MSCs

For peripheral delivery of hUC-MSCs, 1 × 10^7^ cells were suspended in 100 ml normal saline, and the infusion rate of peripheral blood was 35–45 drops/min. It took about 50–60 min to complete each 1 × 10^7^ cell infusion.

### Statistical analysis

All data was stated as mean (± s.e.m.). Statistical analysis was performed using GraphPad 5.0 or SPSS 13.0 software. Survival was analyzed using a Kaplan–Meier plot and log-rank analysis. Statistical evaluation of two groups was performed using Student *t* test or Mann–Whitney *U* test if data were not normally distributed. A value of *P* < 0.05 was considered statistically significant.

Other materials and methods are available in the Additional file 1: Supplemental Materials and Methods.

## Results

### Rhesus monkeys immunologically tolerate human umbilical cord mesenchymal stem cells

To evaluate whether monkeys tolerate the xenogenic human cells, we conducted 3 consecutive days of hUC-MSC infusion (1 × 10^7^ cells/monkey/day) (Additional file 1: Figure S2A and Additional file 1: Table S1). We did not observe any prominent changes in the immune system in the monkeys after hUC-MSC infusion, including the numbers of peripheral lymphocytes, monocytes, and neutrophiles; the ratio of CD4+/CD8+ T cells, and the proportion of regulatory T cells and mature dendritic cells (Additional file 1: Figure S2 B-F). In addition, serum levels of IgA, IgG, and IgM were kept stable (Additional file 1: Figure S2G). Our data demonstrated the extremely low immunogenicity of hUC-MSCs and the xenogeneic cell transplantation is safe for monkeys.

### Peripheral delivery of hUC-MSCs prevents ALF development in monkeys

**Fig. 1:**
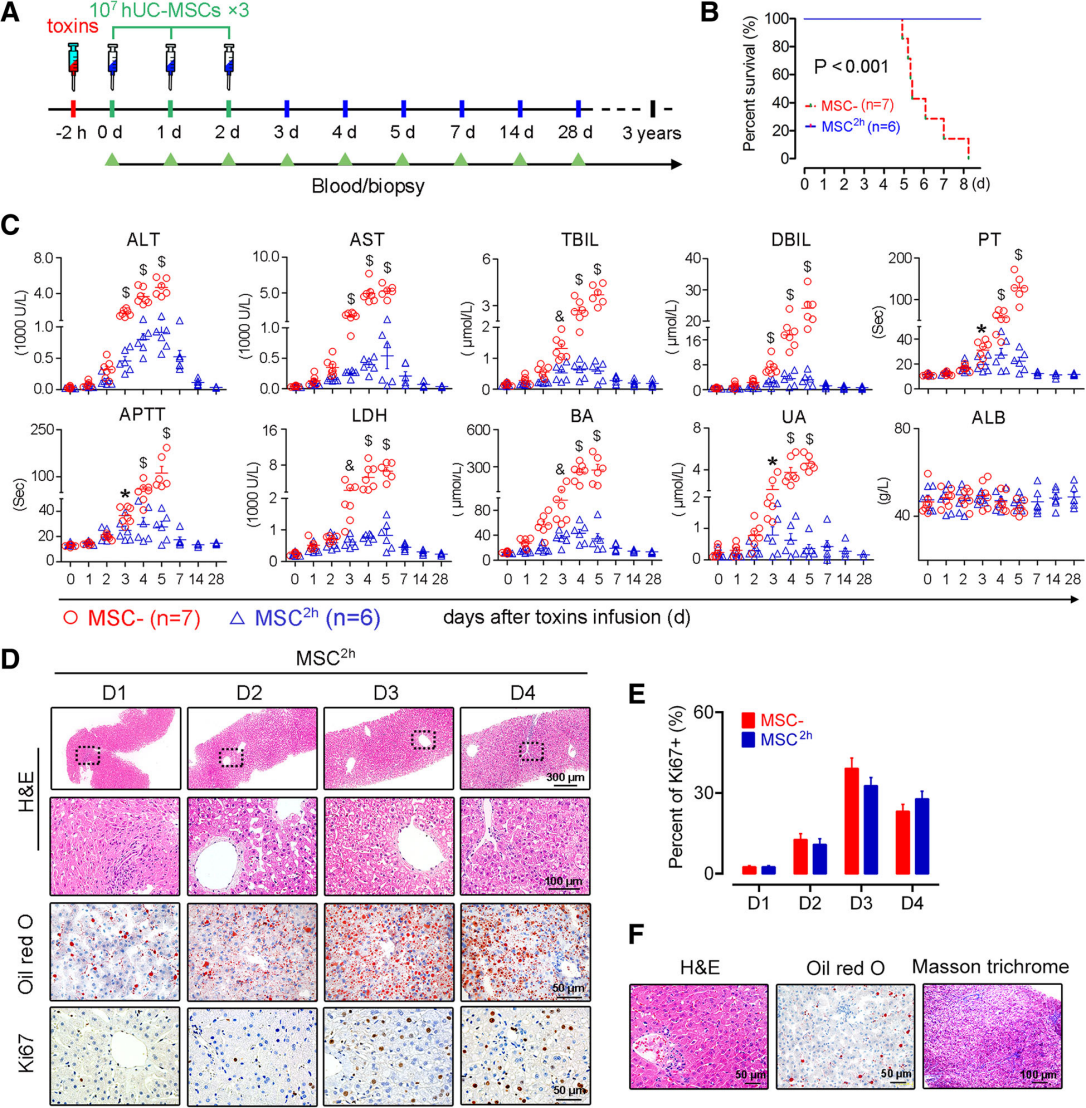
Peripheral delivery of hUC-MSCs ameliorates liver injury and rescues monkeys from lethal ALF. **a** Schematic representation of the experimental design. **b** Survival curves for the monkeys submitted to different treatments (Kaplan–Meier method with log-rank test). **c** Biochemical assays of hepatic indexes: alanine aminotransferase (ALT), glutamic-oxaloacetic transaminase (AST), total bilirubin (TBIL), direct bilirubin (DBIL), prothrombin time (PT), activated partial thromboplastin time (APTT), lactate dehydrogenase (LDH), blood ammonia (BA), uric acid (UA), and albumin (ALB). Error bars, SEM. Mann–Whitney *U* test, *P < 0.05, ^&^*P* < 0.01, and ^$^*P* < 0.001. **d** HE staining, Oil red O staining, and Ki67 immunohistochemical staining of liver specimens at indicated days. **e** Quantitation of Ki67+ positive hepatocytes at indicated time points following different treatments. The numbers of positive cells in 10 consecutive high-power fields were counted. Student’s *t* test, each bar represents the mean ± s.e.m. of positive cells in liver sections from at least three monkeys. **f** Histology of biopsied liver specimens obtained from hUC-MSC-treated monkeys after 2 months of follow-up

Thirteen adult rhesus monkeys were intraperitoneally administered with toxins. The monkeys were assigned randomly into two groups based on gender and age: one group (*n* = 6) was repeatedly peripherally infused with 1 × 10^7^ hUC-MSCs and another group (*n* = 7) received an equal volume of saline at 2 h, 24 h (day 1), and 48 h (day 2) after challenge with the toxins (Fig. 1a and Additional file 1: Table S1). Liver biopsies and blood collections were conducted at indicated time points (Fig. 1a). After a latent period (2 days) following the toxin injection, the saline-treated monkeys demonstrated progressively deteriorated symptoms and signs including poor appetite and vomiting, grasping disability, torpidity, drowsiness, mental indifference, asterixis, and finally, hepatic coma. The monkeys were euthanized when a deep coma developed (Fig. 1b). Serum indices, including liver enzymes, alanine aminotransferase (ALT) and glutamic-oxaloacetic transaminase (AST), blood ammonia (BA), bilirubin (BIL), and clotting time (prothrombin time (PT) and activated partial thromboplastin time (APTT)), were kept stable or slightly increased before day 2 and then continued to rise and increased by dozens to hundreds of folds (Fig. 1c). The biopsies showed that the livers developed apparent steatosis and mild focal necrosis before day 2 and that severe steatosis, patchy and piecemeal necrosis, and sinusoid dilation and hemorrhage appeared at later times [9].

In contrast, the monkeys that received hUC-MSC infusions consistently maintained good physical and mental conditions and achieved long-term survival (Fig. 1b and Additional file 1: Table S1). Before day 2, the levels of serum indicators were not significantly different compared to those of the monkeys that received saline injection. Then, they increased moderately in the hUC-MSC-treated animals, peaking between days 4 and 5 before returning to normal levels within approximately 2 weeks (Fig. 1c). Despite the clear presence of steatosis and a small amount of necrosis in the hepatocytes, the liver structure was fairly well maintained throughout the experimental period (Fig. 1d). In follow-up biopsies, the livers displayed a normal architecture without noticeable degeneration or fibrosis (Fig. 1e). Moreover, neither intrahepatic nor extrahepatic tumors were observed in the recipients during a 3-year follow-up period (data not shown).

### hUC-MSCs neither protect the liver from toxin damage, promote liver repair, nor regulate adaptive immune responses

Because amatoxin was cleared within 24 h [24–26] and because a similar degree of liver damage was observed in both groups before day 2, the hUC-MSCs did not appear to protect the hepatocytes from toxin-induced injury. In addition, the hUC-MSCs were unlikely to have differentiated into hepatocytes within such a short period of time, let alone that few cells of peripheral origin could localize in the liver because we failed to detect superparamagnetic iron oxide (SPIO)-labeled hUC-MSCs using magnetic resonance imaging (MRI) or observe fluorescently labeled cells in liver specimens under fluorescent microscope (data not shown). We next investigated whether hUC-MSCs promoted liver repair. Immunohistochemical staining of Ki67 in the biopsy tissues showed that the surviving hepatocytes were actively proliferating in all of the monkeys during the first 4 days regardless of their intervention. Interestingly, the saline-treated monkeys presented a little higher proliferation indexes during the early stage (Fig. 1d, e).

**Fig. 2:**
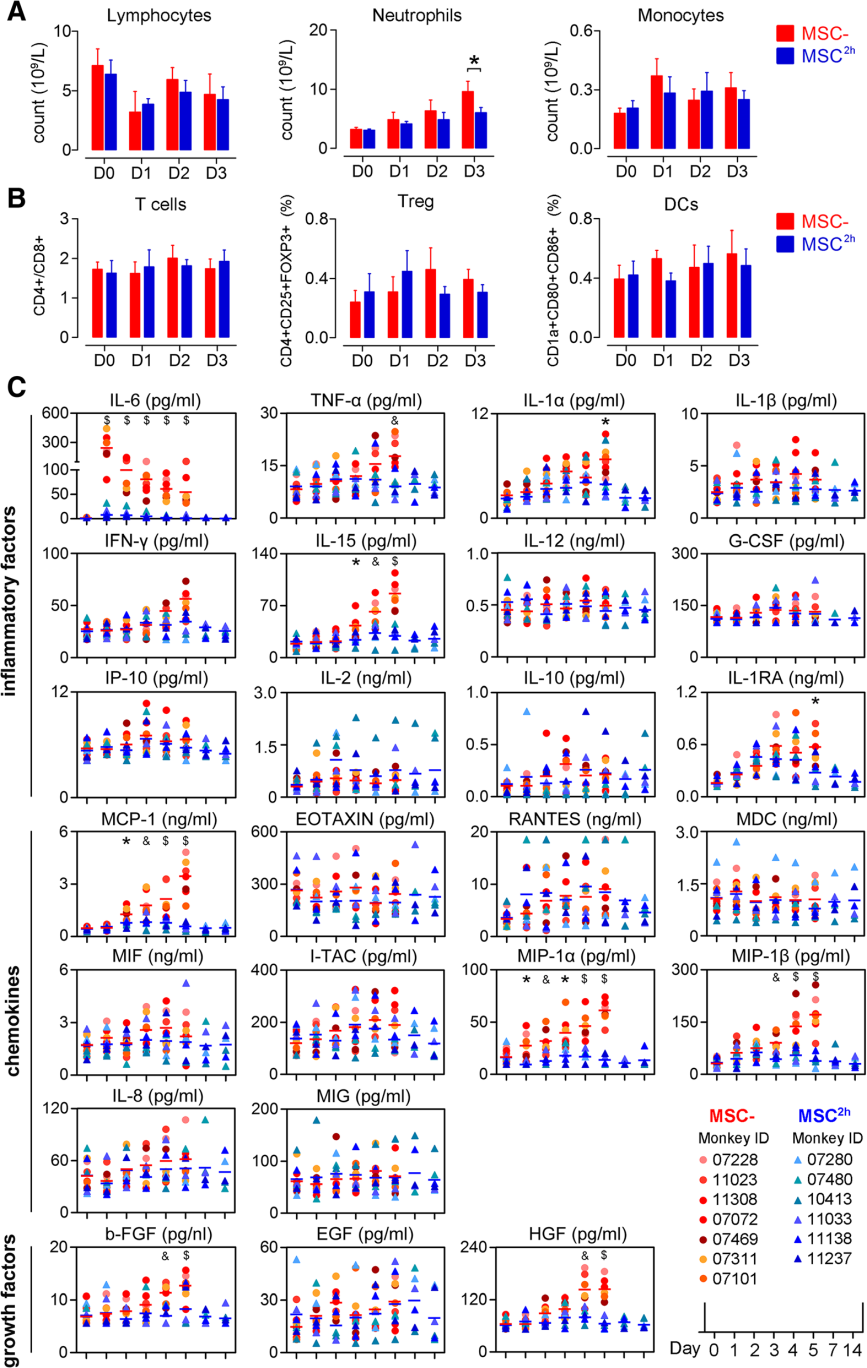
hUC-MSCs suppress systemic inflammation. **a** Count of circulating lymphocytes, neutrophils, and monocytes. **b** Ratio of CD4+/CD8+ T cells and proportion of circulating regulatory T cells (Treg; CD4+CD25+FOXP3+) and dendritic cells (DCs; CD1a+CD80+CD86+). **c** Serum levels of cytokines, chemokines, and growth factors. EGF, epidermal growth factor; eotaxin, eosinophil chemotactic factor; FGF, fibroblast growth factor; G-CSF, granulocyte colony-stimulating factor; HGF, hepatocyte growth factor; IL, interleukin; INF-γ, interferon γ; IP-10, interferon-inducible protein-10; I-TAC, interferon-inducible T cell α chemoattractant; MCP-1, monocyte chemoattractant protein-1; MDC, macrophage-derived chemokine; MIF, macrophage migration inhibitory factor; MIG, monokine induced by interferon γ; MIP-1α, macrophage inflammatory protein-1α; Rantes, regulated upon activation normal T cell expressed and secreted; TNF-α, tumor necrosis factor. *N* = 5–6; error bars, SEM. Mann–Whitney *U* test, *P < 0.05, ^&^*P* < 0.01, and ^$^*P* < 0.001

We did not observe any prominent changes in the components of adaptive immunity in the monkeys after toxin challenge, including the numbers of peripheral lymphocytes, the ratio of CD4+/CD8+ T cells, the proportion of regulatory T cells and mature dendritic cells (Fig. 2a, b and Additional file 1: Figure S3), and the levels of immunoglobulins (Additional file 1: Figure S4). It appeared that the adaptive immune response plays minor role in the pathophysiologic process of ALF and the hUC-MSCs did not modulate antibody- or cell-mediated immune reactions to protect the liver from immune injuries.

### hUC-MSC infusion suppresses systemic inflammation

Because systemic inflammatory reaction syndrome (SIRS) acts a critical role in initiating and accelerating ALF [5, 27], we next investigated whether peripheral infusion of hUC-MSCs can suppress the cytokine storm. We assessed the levels of circulating inflammatory factors. In the saline-treated monkeys, the levels of most factors including the pro-inflammatory cytokines such as TNF-α, IL-1α, IL-1RA, and IL-15; the chemokines such as MCP-1, MIP-1α, and MIP-1β; and the growth factors such as HGF and b-FGF began to steadily increase on day 2 or later and reached their peak levels before the animals were sacrificed. In contrast, in the hUC-MSC-treated monkeys, these factors consistently remained at baseline levels or displayed a slight increase. Most noticeably, in the saline-treated monkeys, we observed a 30- to 160-fold increase in IL-6 as early as 1 day after toxin injection. However, in the hUC-MSC-treated monkeys, IL-6 levels increased only slightly throughout the experimental period (Fig. 2c). Other factors, including INF-γ, IL-1β, IL-2, IL-8, IL-10, IL-12, G-CSF, EGF, Rantes, eotaxin, MIF, I-TAC, MDC, MIG, IP-10, and EGF, also variably increased, but there was no significant difference in their levels between the two groups (Fig. 2c).

### hUC-MSC infusion inhibits the activation of circulating monocytes

**Fig. 3:**
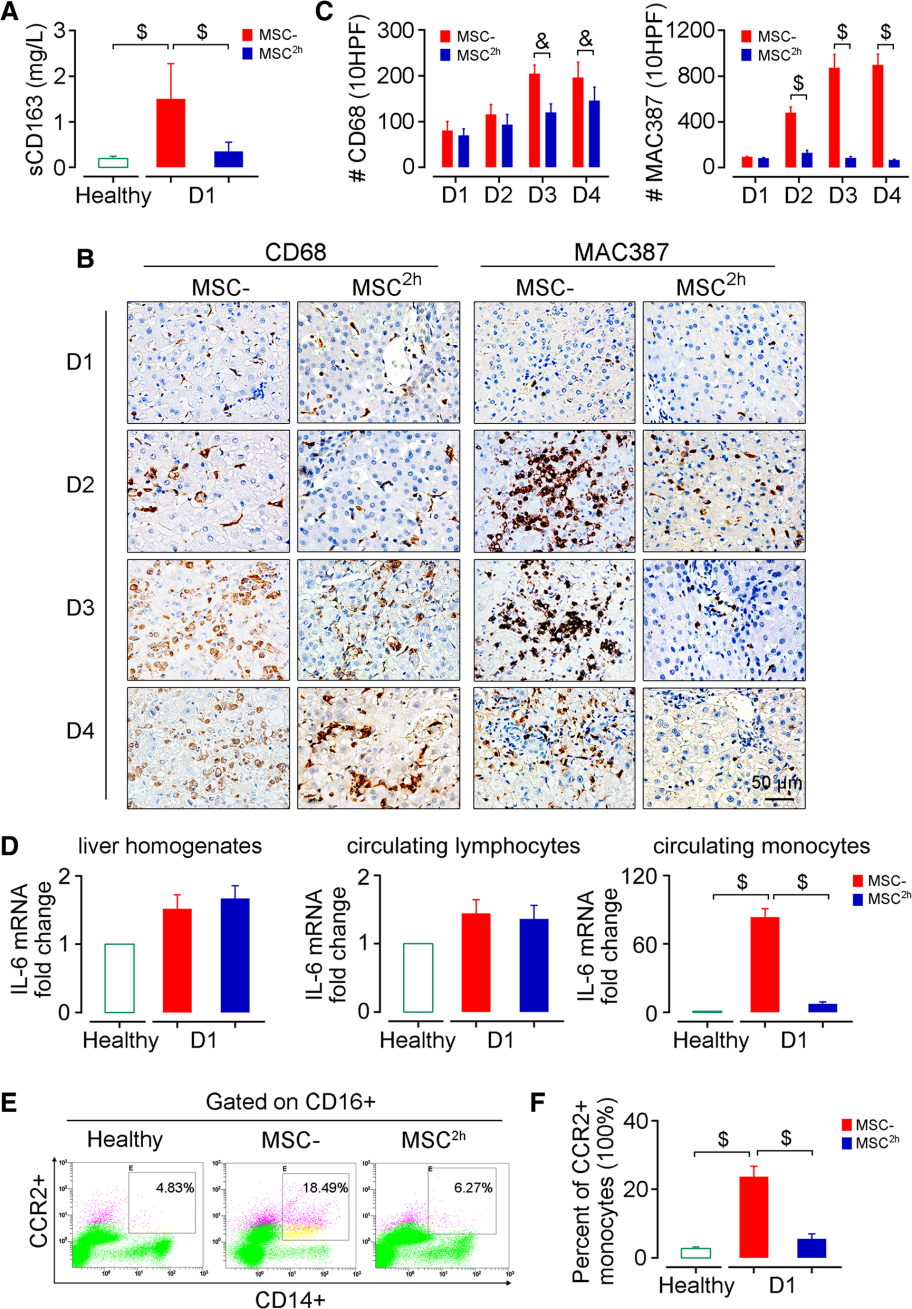
hUC-MSC infusion blocks ALF development by inhibiting the activation of circulating monocytes. **a** ELISA measurement of serum levels of sCD163 in the treated monkeys. *N* = 6. **b** Representative photomicrographs of the results of immunohistochemical staining to identify resident KCs (CD68+) and recruited hepatic macrophages (MAC387+) in liver tissues. **c** Quantitation of CD68+ and MAC387+ macrophages. The numbers of CD68+ and MAC387+ cells were counted in 10 consecutive high-power fields in three monkeys. For each section, 10 randomly chosen portal tracts or areas of central veins were assessed at high magnification (× 400), and the cumulative number of positive cells in 10 HPF was recorded. **d** Transcript levels of IL-6 in liver homogenates, circulating monocytes, and lymphocytes. Each bar represents data obtained from at least three independent quantitative PCR experiments. **e**, **f** Flow cytometry analysis of activated monocytes (CD14+CD16+CCR2+) in subsets of peripheral blood monocyte, *N* = 6. Error bars, SEM. Student’s *t* test, *P < 0.05, ^&^*P* < 0.01, and ^$^*P* < 0.001

As a pro-inflammatory cytokine, IL-6 targets numerous genes that are known to regulate liver repair following a diverse array of injuries [28]. However, excessive production of IL-6 also acts a critical role in the initiation and promotion of systemic inflammation. We have revealed that the activated c-Mos, but not resident Kupffer cells, act an essential role in IL-6 producing after toxin challenge [9], and we then assessed whether infusion of hUC-MSCs suppresses c-Mos activation. As reported previously [9], in the saline-treated monkeys, with the increase of IL-6, serum levels of soluble CD163 (sCD163), which is released uniquely by activated macrophages [29], were over 100-fold increased at day 1 (Fig. 3a). In contrast, the levels of sCD163 were extremely suppressed in the hUC-MSC-treated monkeys. Unexpectedly, although CD68 staining increased substantially with the progression of ALF, we did not observe noticeable aggregation of CD68+ cells in the biopsy tissues before day 2 (Fig. 3b, c). The proportion of activated monocytes, characterized by CD14+CD16+CCR2+ [7, 30], increased substantially in the saline-treated monkeys, and these cells expressed much higher levels of IL-6 mRNA (Fig. 3d–f). Furthermore, a great number of c-Mos, characterized by positive MAC387 immunohistochemistry staining [31], began to recruit in the liver and differentiate into mature macrophages at day 2 (Fig. 3b, c). On the contrary, the activation and hepatic recruitment of c-Mos were both strongly suppressed in the hUC-MSC-treated monkeys (Fig. 3b, c). Most noticeably, the proportion of CD14+CD16+CCR2+ monocytes was reduced substantially in these animals (Fig. 3d–f).

### Delayed hUC-MSC treatment improves outcomes in monkeys challenged with toxins

**Fig. 4:**
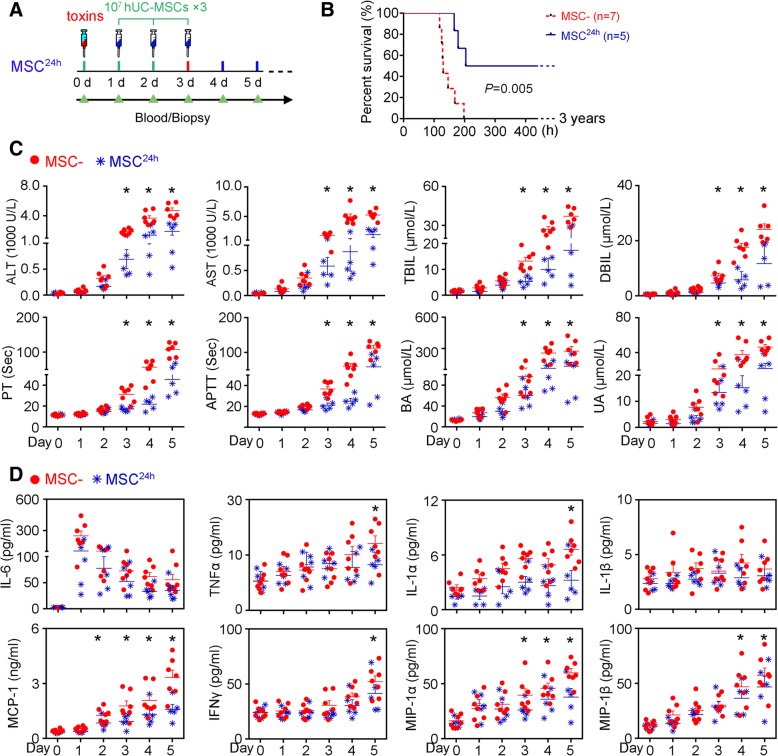
Delayed hUC-MSC treatment improves outcomes in monkeys challenged with toxins. **a** Schematic representation of the experimental design. **b** Survival curves for the monkeys submitted to different treatments (Kaplan–Meier method with log-rank test). **c** Biochemical assays of hepatic indexes: alanine aminotransferase (ALT), glutamic-oxaloacetic transaminase (AST), total bilirubin (TBIL), direct bilirubin (DBIL), prothrombin time (PT), activated partial thromboplastin time (APTT), blood ammonia (BA), and uric acid (UA). Error bars, SEM. Mann–Whitney *U* test, *P < 0.05. **d** Serum levels of cytokines, chemokines, and growth factors. IL, interleukin; TNF-α, tumor necrosis factor; MCP-1, monocyte chemoattractant protein-1; INF-γ, interferon γ; MIP-1α, macrophage inflammatory protein-1α; MIP-1α, macrophage inflammatory protein-1β. *N* = 5–7; error bars, SEM. Mann–Whitney *U* test, *P < 0.05

Our data suggested that early infusion of hUC-MSCs disrupts the inflammatory cascade by inhibiting the overproduction of monocyte-derived IL-6. However, in humans, patients seek medications only when their symptoms become apparent, with their systemic homeostasis already severely impaired. We therefore assessed whether treatment with hUC-MSCs improves prognoses in monkeys with fully developed ALF. We began to infuse the monkeys with hUC-MSCs at 24 h after injection of the toxins (Fig. 4a), since serum IL-6 cytokine levels reached maximum at this time point and circulating mononuclear cells also have been highly activated and expressed IL-6 mRNA according to our previous evidence [9] (Figs. 2c and 3). Although only two of the five monkeys achieved complete recovery, the other three monkeys died during the 166–204-h period following toxin challenge (Fig. 4b and Additional file 1: Table S1), treatment with hUC-MSCs significantly improved their hepatic indices (Fig. 4c). Notably, in spite that IL-6 exhibited no significant difference between two groups throughout the experimental period, other factors, such as MCP-1, MIP-1α, and MIP-1β, were reduced following hUC-MSC infusion (Fig. 4d).

### hUC-MSCs inhibit monocyte activation in vitro

**Fig. 5:**
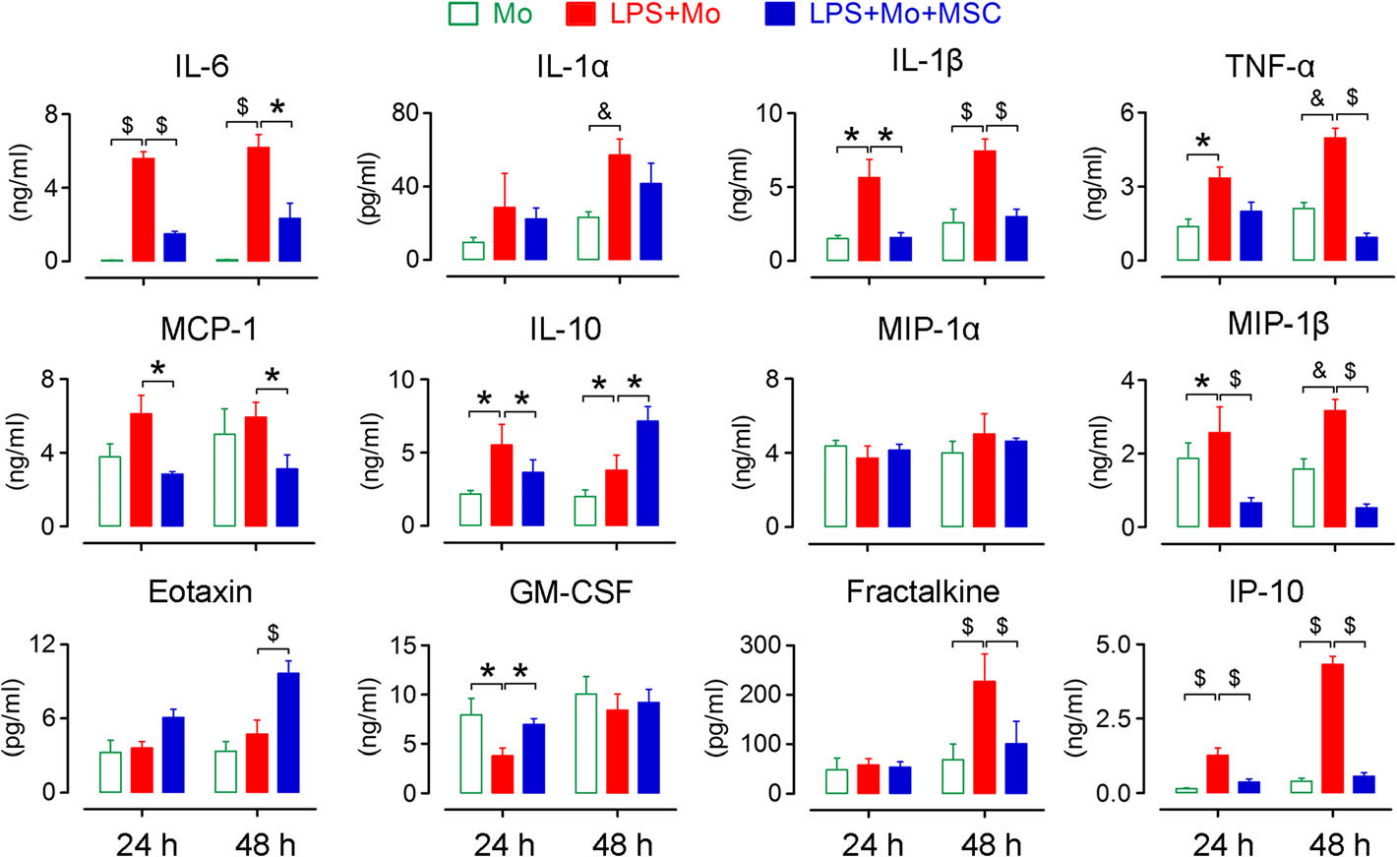
hUC-MSCs inhibit the activation of monocytes in vitro. hUC-MSCs suppressed the production of pro-inflammatory factors by monocytes in co-cultures. IL, interleukin; TNF-α, tumor necrosis factor; MCP-1, monocyte chemoattractant protein-1; MIP-1α, macrophage inflammatory protein-1α; MIP-1α, macrophage inflammatory protein-1β; GM-CSF, granulocyte–macrophage colony-stimulating factor; IP-10, interferon-inducible protein-10. *N* = 6; error bars, SEM. Student’s *t* test, *P < 0.05, ^&^*P* < 0.01, and ^$^*P* < 0.001

To further confirm that hUC-MSCs suppress the activation of c-Mos, we isolated quiescent monocytes from healthy monkeys and stimulated them with LPS for activation [32]. After 24 h or 48 h of cultivation, the concentrations of the pro-inflammatory factors such as IL-6, TNF-α, MCP-1, IL-1α, IL-1β, MIP-1β, and IP-10 increased markedly in the culture medium. When monocytes were co-cultured with hUC-MSCs in a transwell system, the concentrations of these cytokines were significantly lower in the medium (Fig. 5). These data suggested that hUC-MSCs were actually potent to suppress the activation of macrophages. Most strikingly, in contrast to most of the inflammatory factors that were inhibited by the co-culture of hUC-MSC, IL-10, a well-known inhibitory factor, was significantly increased after 48-h co-culture.

### hUC-MSCs are likely to express inhibitory genes to suppress monocyte activation

**Fig. 6:**
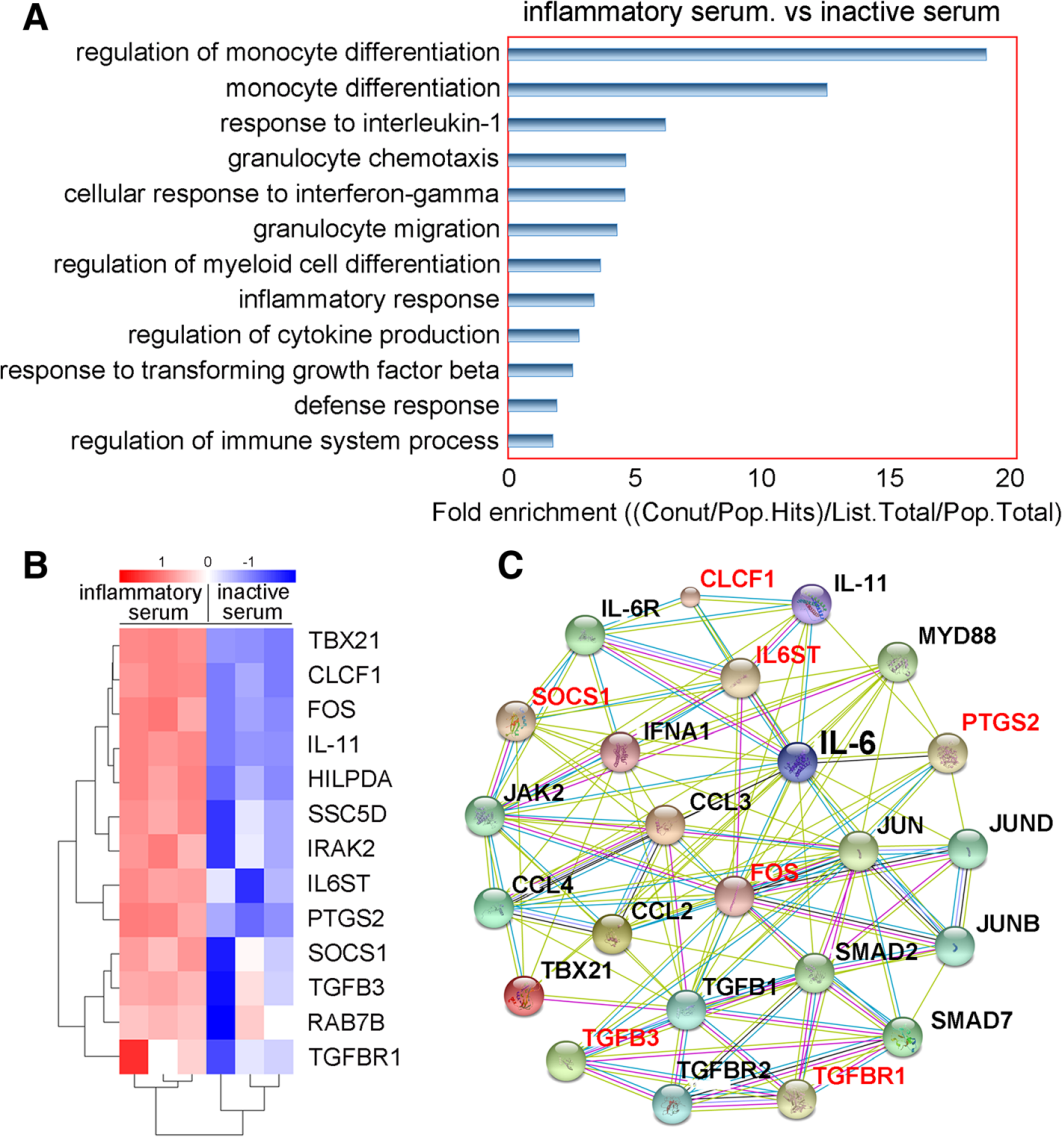
hUC-MSCs produce inhibitory factors in response to inflammatory stimulation. **a** Gene ontology term analysis of the primary altered genes that are associated with the regulation of inflammatory processes. *N* = 3. **b** Heat map of the upregulated genes identified in the microarray analysis. *N* = 3. **c** Ingenuity pathway analysis of the network of upregulated inhibitory genes (in red) in hUC-MSCs stimulated by inflammatory serum

The in vivo mechanism by which hUC-MSCs inhibit the activation of c-Mos and the production of IL-6 remains unclear because we could not analyze the activities of the infused hUC-MSCs. Similarly, in the co-culture system, we could not determine whether the hUC-MSCs secreted anti-inflammatory cytokines, such as IL-10, or stimulated the monocytes themselves to produce such factors. To test the hypothesis that hUC-MSCs adaptively produce inhibitory cytokines in response to a pro-inflammatory microenvironment, we stimulated cultured hUC-MSCs using inflammatory monkey serum (i.e., serum isolated on day 1 following toxin challenge) or inactive serum (i.e., serum isolated from healthy monkeys). After a 30-min stimulation, the cells were collected for microarray gene expression profiling. The results revealed that a total of 188 genes were upregulated while 433 genes were downregulated in hUC-MSCs (Additional file 1: Figure S5; ArrayExpress accession number: E-MTAB-4750, https://www.ebi.ac.uk/arrayexpress/experiments/E-MTAB-4750/). We further analyzed the profiles of the upregulated genes to identify the networks and biological processes involved in the changes. We found that components of molecular networks associated with inflammatory responses, defenses, monocyte differentiation, and myeloid cell differentiation were upregulated (Fig. 6a). Interestingly, the inhibitory genes Fos, Socs1, Ssc5d, Zbtb46, Tgfb3, Rab7b, and Tgfbr1, which negatively regulate cytokine signaling and inflammatory cell differentiation, inhibit inflammatory activity, or have a role in immunosuppression, were all found to be expressed at significantly higher levels (Fig. 6b, c).

## Discussion

The development of efficient therapeutics for ALF has been hindered by the unclear pathogenesis [1]. In this study, by using a large non-human primate model of ALF, we found that c-Mos and their product IL-6 play critical roles in initiating and accelerating ALF development [9]. The early peripheral infusion of hUC-MSCs profoundly suppressed the activation of c-Mos and has improved markedly the hepatic histology, systemic homeostasis, and animal survival.

Literatures suggest disruption of the immune system, especially that the dysregulation of the innate immune system plays a critical role in the development of ALF [5, 8, 33, 34]. Within the innate immune system, Kupffer cells (KCs) are the first cell groups which will receive the “alarm” signals following primary hepatocyte injury [8]. The activated KCs will then produce various cytokines and chemokines to activate or recruit other inflammatory cells resulting in systemic release of cytokines, growth factor, and chemokines to restore homeostasis. At the same time, the hypercytokinemia, also described as “cytokine storm,” will accelerate the rate of tissue damage and SIRS [5, 7, 34]. The immune paralysis in consequent of uncontrolled SIRS is closely associated with recurrent sepsis and multi-organ dysfunction [5]. Upon liver uptake, amanitin will be fully cleared within 24 h but we did not notice apparent hepatocyte necrosis within 48 h. Therefore, the mild primary liver injury can result in severe local tissue damage and systemic disarrangement, highlighting the crucial role of the secondary systemic inflammation in promoting ALF.

We have previously demonstrated that the level of IL-6 was the most immediately and dramatically increased cytokine after toxin infusion. As a pro-inflammatory cytokine, IL-6 targets numerous immediate-early genes which regulate liver repair mechanism following multiple hepatic injuries. Nevertheless, increased expression of IL-6 often leads to uncontrolled inflammatory reaction and tissue damage [35, 36]. In contrast to the previous findings which report that IL-6 is primarily produced by KCs [5, 28, 35], we did not find significant activation and expansion of KCs within 48 h following toxin exposure. In contrary, we found that c-Mos were the major IL-6 producers before they recruited to the liver and differentiate into mature macrophages, making c-Mos as a promising target in developing treatment for ALF.

The clinical application of MSCs to treat ALF is controversial because of the underlying pathophysiology mechanism of ALF, and functions of MSCs have not yet been elucidated [18, 37]. We have excluded the possibility that hUC-MSCs promote liver repair, differentiate into hepatocytes, or protect the injured liver by modulating the adaptive immunity. Many cytokines were suppressed, particularly the decreased serum chemokines including MCP-1, MIP-1α, and MIP-1β, which suggested that peripherally delivered hUC-MSCs inhibit the activation of monocytes. The mechanism by which hUC-MSCs interact with c-Mos remains unclear. It has been established that IL-10 promotes the transition of macrophages from the M1 to M2 phenotypes [17]. Our results did not directly support the fact that hUC-MSCs produce IL-10 to suppress the activation of c-Mos. Nevertheless, in an in vitro experiment, when stimulated with inflammatory serum, hUC-MSCs adaptively produce inhibitory factors that suppress the activation of innate immune cells and inflammatory responses. In addition, the peripherally infused hUC-MSCs have more opportunities to interact with c-Mos.

The potential immunogenicity and tumorigenicity of hUC-MSCs need to be further investigated before translated into clinical applications [19, 37]. The study found that rhesus monkeys tolerated the xenogeneic human cells, confirming their low immunogenicity and suggesting that it may be feasible to use hUC-MSCs in allogenic recipients. Moreover, during the 3-year follow-up period, we found no evidence of any tumors in the animals that received hUC-MSCs. Thus, hUC-MSCs appear to be safe for a clinical setting.

The main limitations of this study are the low quantity of cell count relative to the large size of the animal. This caused difficulty in tracking and determining peripherally infused cells. Due to the high cost of primates, we were unable to determine an optimal time frame for cell infusion. Our findings support the implementation of early cell therapy prior to the full development of a cytokine storm. Nevertheless, even when the optimal therapeutic window for this treatment is missed in a clinical setting, it still remains a valuable option. Finally, we did not assess the dose–effect relationship. It is reasonable to speculate that infusing more cells would be more effective, particularly in patients with fully fueled systemic inflammation, which requires further evaluation.

## Conclusions

**Fig. 7:**
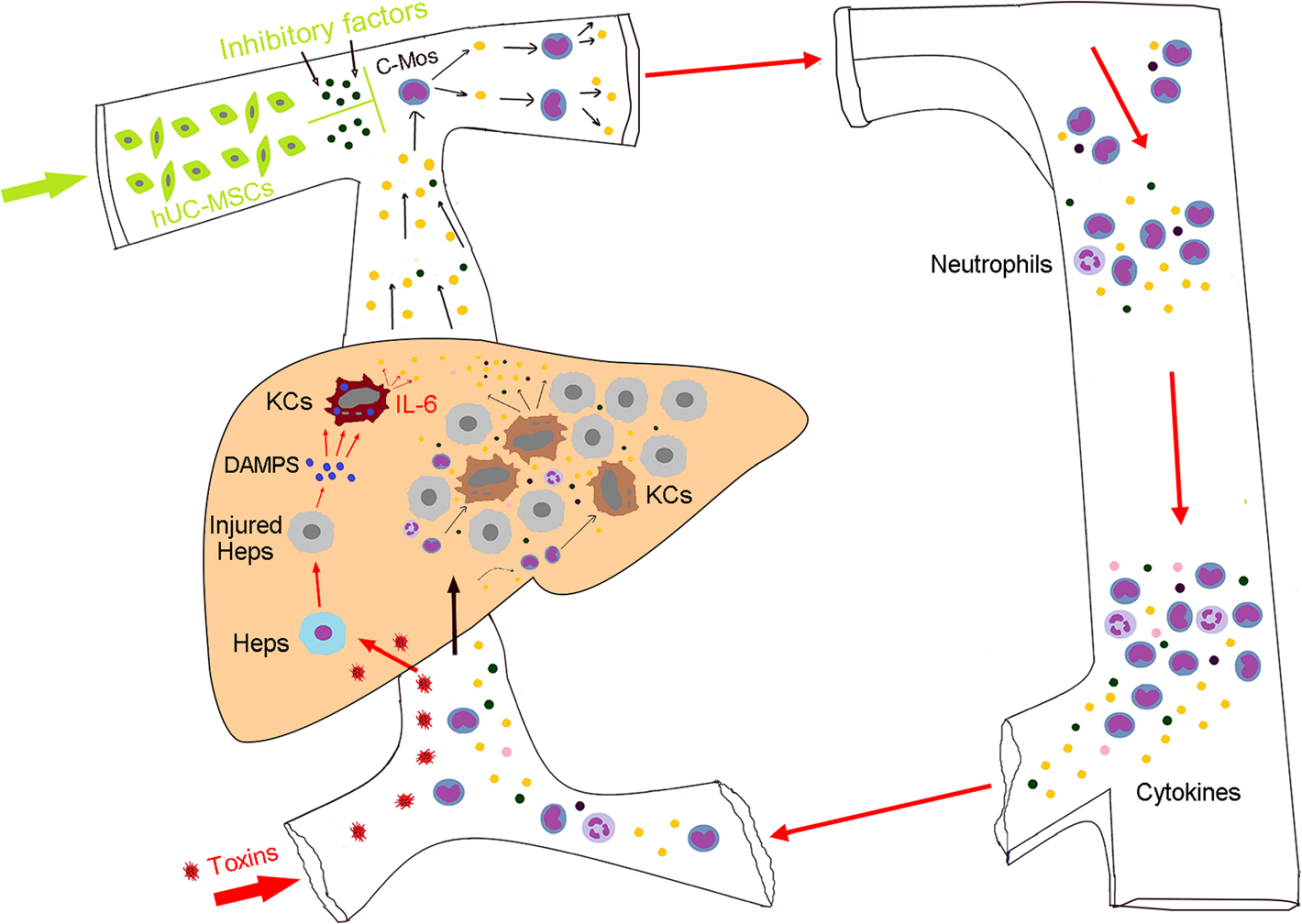
The hypothetical pathway through which hUC-MSC infusion disrupts the development of ALF. Toxin injection destroys hepatocytes (HC), inducing the release of damage-associated molecular patterns (DAMPs), which activate resident Kupffer cells (KC). KCs then release cytokines including IL-6, fueling the circulating monocytes (c-Mos) to secret more IL-6 and activate more c-Mos, and as a result, cytokine storm occurs. Meanwhile, the activated c-Mos migrate to the liver and differentiate into mature macrophages (MФ), which further augment the tissue damage. Infusion of hUC-MSCs suppresses the activation of c-Mos, thereby disrupting the over activation of inflammatory cascade and the deterioration of systemic disarrangement

We used a large, non-human primate model to provide novel insights of the underlying cellular and molecular events of ALF pathogenesis. Our work demonstrates that peripheral infusions of hUC-MSCs profoundly suppressed the activation of c-Mos, which resulted in the prevention of the development of lethal ALF in monkeys (Fig. 7). These results, in combination with those demonstrating the efficacy and safety approach, indicate that hUC-MSC-based therapies are promising strategy which need further investigations and validation before being applied in clinical practice.

## Additional file

Additional file 1: **Table S1.** General information and treatments of monkeys. Table S2. hUC-MSCs quality inspection report: viral factors and pathogens. Figure S1. The surface markers and multiple differentiation potentialities of hUC-MSCs. (A) Flow cytometric analysis of the cell markers of mesenchymal stem cells. (B) Adipogenic differentiation and osteogenic differentiation of hUC-MSCs were determined by Oil red O staining and alizarin red staining. Scale bar = 50 μm. Figure S2. The rhesus monkeys completely tolerated the xenogeneic hUC-MSCs. (A) Schematic representations of the experimental designs. (B) Sum and sort counting of leukocytes. (C, D) Flow cytometric analysis and quantitation of the ratio of the CD4+/CD8+ cells. (E, F) Flow cytometric analysis and quantitation of the proportion of CD4+CD25+FoxP3+ regulatory T cells. (G) Serum levels of IgA, IgG, and IgM over time. Each bar represents the mean ± s.e.m., *n* ≥ 3. Figure S3. hUC-MSC infusion does not change peripheral leukocytes, ratio of CD4+/CD8+ T cell, regulatory T cells and DCs. (A) Flow cytometric analysis and quantitation of the ratio of CD4+/CD8+ T cells. (B) Flow cytometric analysis of CD4+/CD25+FoxP3+ regulatory T cells. (C) Flow cytometric analysis of CD1a+/CD80+/CD86+ dendritic cells. Each bar represents the mean ± s.e.m., *n* ≥ 5/group. Figure S4. hUC-MSC infusion does not disturb antibodies and complements. Student’s *t* test, the data are presented as the means±s.e.m., *n* ≥ 5. Figure S5. Heat map of altered genes from microarray analysis. ArrayExpress accession number: E-MTAB-4750, https://www.ebi.ac.uk/arrayexpress/experiments/E-MTAB-4750/. (DOCX 3772 kb)

## Abbreviations

ALB: Albumin
ALF: Acute liver failure
ALT: Alanine aminotransferase
APTT: Activated partial thromboplastin time
AST: Glutamic-oxaloacetic transaminase
BA: Blood ammonia
CCR2: C-C chemokine receptor type 2
CD: Cluster of differentiation
c-Mos: Circulating monocytes
DBIL: Direct bilirubin
ECF: Eosinophil chemotactic factor
EGF: Epidermal growth factor, eotaxin
FGF: Fibroblast growth factor
G-CSF: Granulocyte colony-stimulating factor
HGF: Hepatocyte growth factor
hUC-MSCs: Human umbilical cord MSCs
Ig: Immunoglobulin
IL: Interleukin
IL-6: Interleukin-6
INF-γ: Interferon γ
IP-10: Interferon-inducible protein-10
I-TAC: Interferon-inducible T cell α chemoattractant
LDH: Lactate dehydrogenase
LPS: Lipopolysaccharide
MCP-1: Monocyte chemoattractant protein-1
MDC: Macrophage-derived chemokine
MIF: Macrophage migration inhibitory factor
MIG: Monokine induced by interferon γ
MIP-1α: Macrophage inflammatory protein-1α
PT: Prothrombin time
Rantes: Regulated upon activation normal T cell expressed and secreted
sCD163: Serum levels of soluble CD163
TBIL: Total bilirubin
TNF-α: Tumor necrosis factor
UA: Uric acid
