# Supplementary material for: Peripheral infusion of human umbilical cord mesenchymal stem cells rescues acute liver failure lethality in monkeys
Source: Stem Cell Res Ther. 2019 Mar 12;10:84. doi: 10.1186/s13287-019-1184-2 (PMC6417089; doi:10.1186/s13287-019-1184-2)
Supplement: Supplementary file 1 — Table S1. General information and treatments of monkeys. Table S2. hUC-MSCs quality inspection report: viral factors and pathogens. Figure S1. The surface markers and multiple differentiation potentialities of hUC-MSCs. (A) Flow cytometric analysis of the cell markers of mesenchymal stem cells. (B) Adipogenic differentiation and osteogenic differentiation of hUC-MSCs were determined by Oil red O staining and alizarin red staining. Scale bar = 50 μm. Figure S2. The rhesus monkeys completely tolerated the xenogeneic hUC-MSCs. (A) Schematic representations of the experimental designs. (B) Sum and sort counting of leukocytes. (C, D) Flow cytometric analysis and quantitation of the ratio of the CD4+/CD8+ cells. (E, F) Flow cytometric analysis and quantitation of the proportion of CD4+CD25+FoxP3+ regulatory T cells. (G) Serum levels of IgA, IgG, and IgM over time. Each bar represents the mean ± s.e.m., n ≥ 3. Figure S3. hUC-MSC infusion does not change peripheral leukocytes, ratio of CD4+/CD8+ T cell, regulatory T cells and DCs. (A) Flow cytometric analysis and quantitation of the ratio of CD4+/CD8+ T cells. (B) Flow cytometric analysis of CD4+/CD25+FoxP3+ regulatory T cells. (C) Flow cytometric analysis of CD1a+/CD80+/CD86+ dendritic cells. Each bar represents the mean ± s.e.m., n ≥ 5/group. Figure S4. hUC-MSC infusion does not disturb antibodies and complements. Student’s t test, the data are presented as the means±s.e.m., n ≥ 5. Figure S5. Heat map of altered genes from microarray analysis. ArrayExpress accession number: E-MTAB-4750, https://www.ebi.ac.uk/arrayexpress/experiments/E-MTAB-4750/. (DOCX 3772 kb) [file 13287_2019_1184_MOESM1_ESM.docx]

**Supplemental Materials and Methods**

**The details of following materials and methods:** Histology staining, Biochemical evaluation, Monkey cytokine level analysis, Assays of sCD163 in monkey serum, Monkey monocyte isolation, Circulating lymphocytes isolation, Flow cytometric assay, mRNA isolation and real-time RT-PCR, please refer to our previous research [[1](#_ENREF_1)].

**In vitro co-culture assay.** For transwell co-culturing, 2×10^5^ monocytes primarily isolated from healthy monkeys were seeded onto a six-well plate. Twenty-four hours later, a 0.4-μm-pore size Corning transwell insert (Sigma-Aldrich) containing 1×10^5^ hUC-MSCs were placed into the six-well plate. Co-cultures were incubated with or without 1 μg/ml LPS for 24 or 48 h. A human cytokine magnetic kit (HCYTOMAG-60K-21, Millipore) was conducted to determine the levels of inflammatory factors in the supernatants of co-culture systems.

**Microarray analysis.** hUC-MSCs were cultured in a T25 culture flasks until 70-80% confluent and then the regular medium (MesenPRO RS^TM^ Medium, Thermo Fisher) was replaced by serum free medium with 10% inflammatory monkey serum collected at day 1 following toxin challenge or heat-inactivated monkey serum. After a 30-min stimulation, cells were harvested for RNA extract and microarray assay. Agilent whole genome microarray assay and data analysis were serviced by Kangchen Bio-tech Inc (Shanghai, China).

[1] Guo G, Zhu Y, Wu Z, Ji H, Lu X, Zhou Y, et al. Circulating monocytes accelerate acute liver failure by IL-6 secretion in monkey. 2018.

**Supplemental Table 1.**

General information and treatments of Monkeys

| Monkey  ID | Sex  (F/M) | Experiment age (years) | Weight  (kg) | Treatment | Survival time  (h) |
| --- | --- | --- | --- | --- | --- |
| 05106 | F | 6.5 | 7.2 | MSC | Long term |
| 10428 | F | 4.5 | 4.7 | MSC | Long term |
| 10243 | M | 5.0 | 5.3 | MSC | Long term |
| 11417 | M | 5.0 | 6.6 | MSC | Long term |
| 11247 | M | 4.5 | 5.8 | MSC | Long term |
| 07082 | F | 6.0 | 7.2 | MSC | Long term |
| 07228 | F | 6.0 | 7.1 | Toxins+NS | 125 |
| 11023 | M | 5.5 | 5.2 | Toxins+NS | 130 |
| 11308 | F | 4.5 | 5.1 | Toxins+NS | 146 |
| 07072 | F | 6.0 | 6.3 | Toxins+NS | 168 |
| 07469 | M | 5.5 | 6.7 | Toxins+NS | 160 |
| 07311 | M | 6.0 | 5.8 | Toxins+NS | 170 |
| 07101 | M | 5.5 | 7.2 | Toxins+NS | 128 |
| 07280 | F | 5.5 | 6.5 | Toxins & MSC^2h^ | Long term |
| 07480 | F | 6.5 | 7.1 | Toxins & MSC^2h^ | Long term |
| 10413 | M | 5.5 | 5.7 | Toxins & MSC^2h^ | Long term |
| 11033 | M | 5.0 | 6.1 | Toxins & MSC^2h^ | Long term |
| 11138 | F | 6.0 | 6.4 | Toxins & MSC^2h^ | Long term |
| 11237 | M | 5.0 | 5.5 | Toxins & MSC^2h^ | Long term |
| 07323 | M | 6.5 | 7.1 | Toxins & MSC^24h^ | 166 |
| 11358 | F | 5.5 | 5.7 | Toxins & MSC^24h^ | Long term |
| 10475 | M | 5.0 | 6.1 | Toxins & MSC^24h^ | Long term |
| 09226 | F | 6.0 | 6.4 | Toxins & MSC^24h^ | 179 |
| 10521 | M | 5.0 | 5.5 | Toxins & MSC^24h^ | 204 |

**Supplemental Table 2.**

hUC-MSCs Quality Inspection report: viral factors and pathogens

|  | Category | Result |
| --- | --- | --- |
| Viral factors | HBsAg | Negative |
|  | Anti-HIV | Negative |
|  | Anti-HCV | Negative |
|  | Syphilis antibody | Negative |
|  | CMV antibody | Negative |
| Pathogens | Aerobic bacteria | Negative |
|  | Anaerobic bacteria | Negative |
|  | Fungi | Negative |
|  | Mycoplasma | Negative |
|  | Bacterial endotoxin | Qualified |


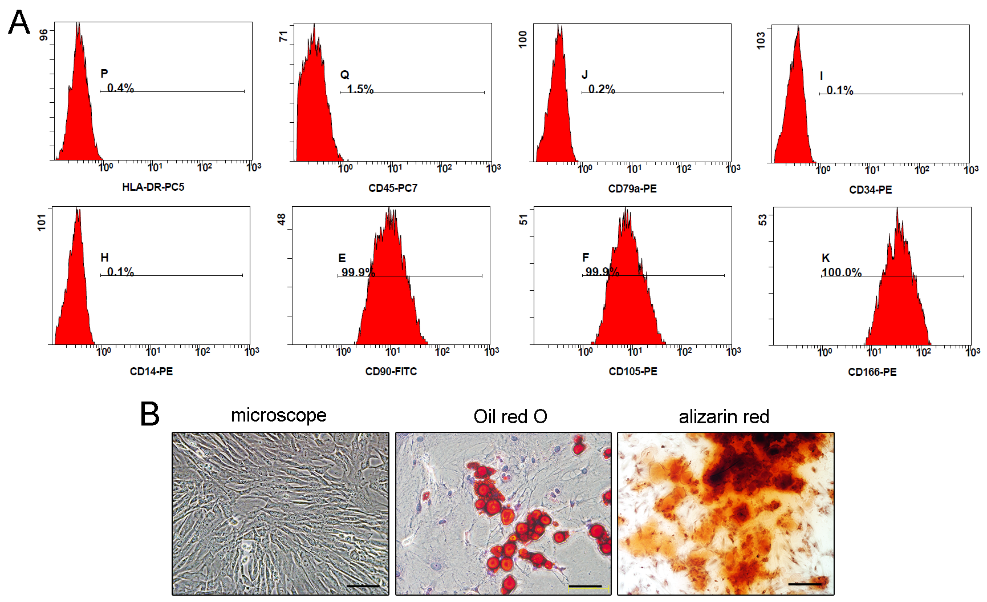


**Supplemental Figure 1.** **The surface markers and multiple differentiation potentialities of hUC-MSCs.** (A) Flow cytometric analysis of the cell markers of mesenchymal stem cells. (B) Adipogenic differentiation and osteogenic differentiation of hUC-MSCs were determined by Oil red O staining and alizarin red staining. Scale bar= 50 μm.


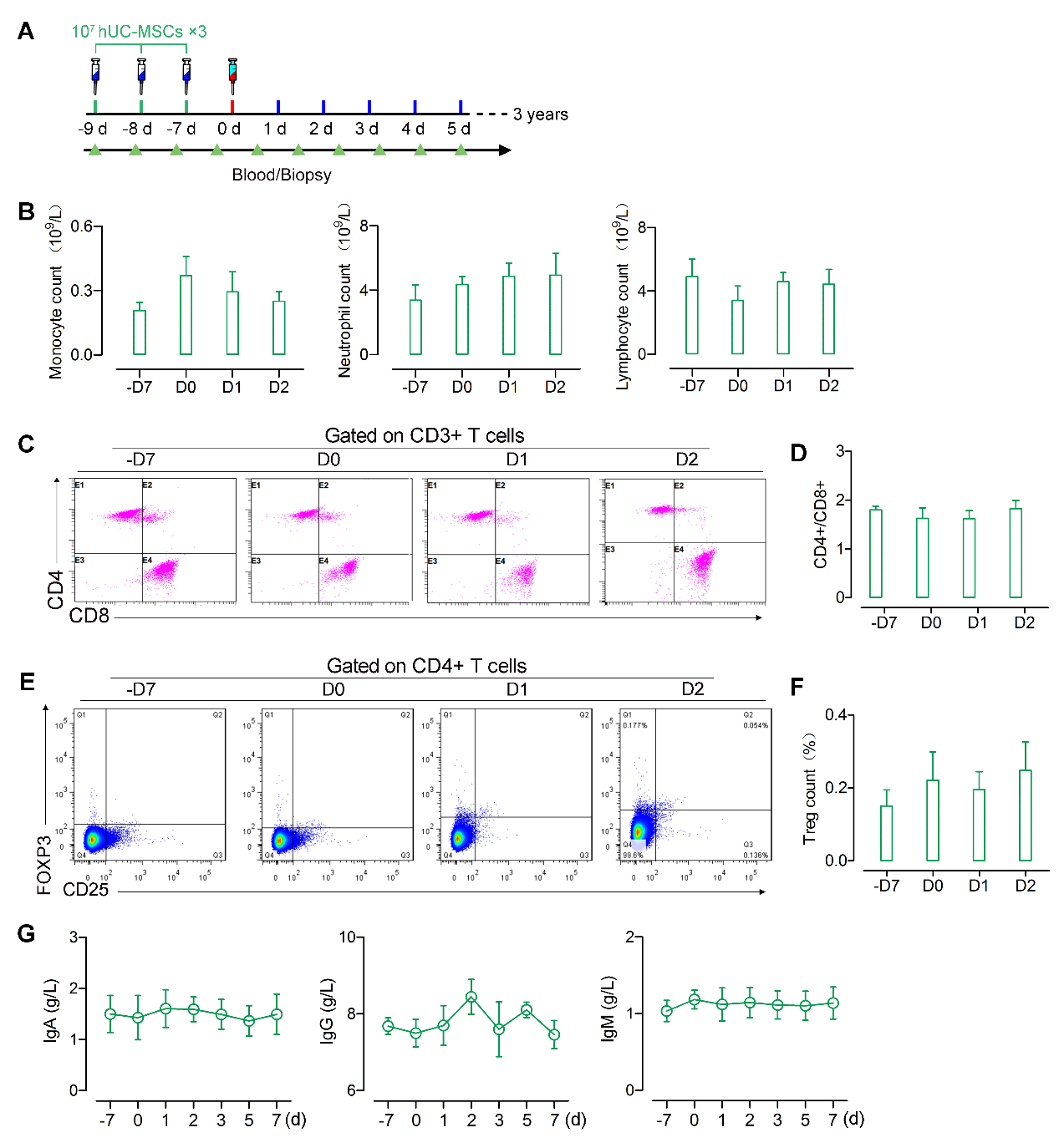


**Supplemental Figure 2. The rhesus monkeys completely tolerated the xenogeneic** **hUC-MSCs.** (A) Schematic representations of the experimental designs. (B) Sum and sort counting of leukocytes. (C, D) Flow cytometric analysis and quantitation of the ratio of the CD4+/CD8+ cells (E,F) Flow cytometric analysis and quantitation of the proportion of CD4+CD25+FoxP3+ regulatory T cells. (G) Serum levels of IgA, IgG, and IgM over time. Each bar represents the mean±s.e.m., n≥3.


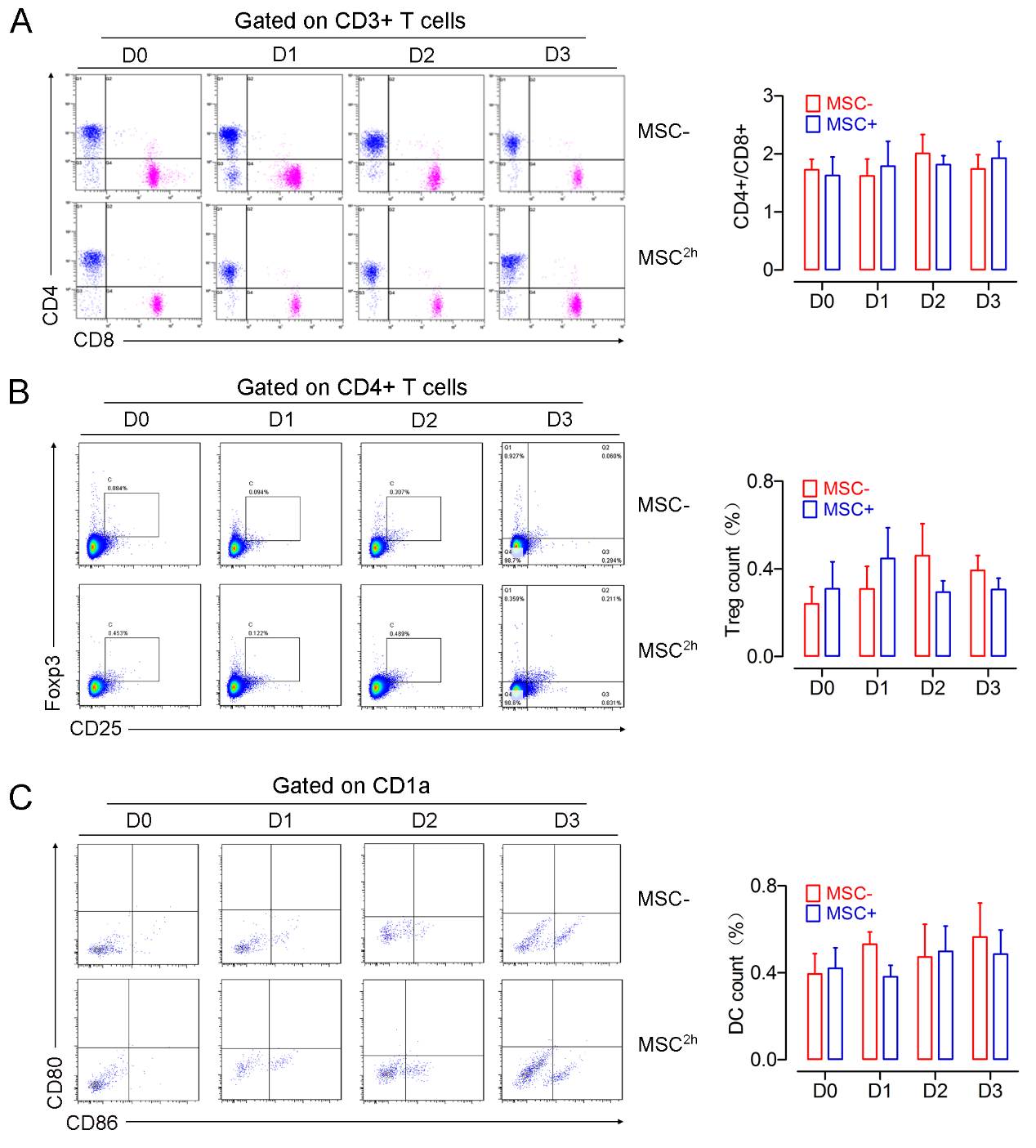


**Supplemental Figure 3.** **hUC-MSC infusion does not change peripheral leukocytes, ratio of CD4+/CD8+ T cell, regulatory T cells and DCs.** (A) Flow cytometric analysis and quantitation of the ratio of CD4+/CD8+ T cells. (B) Flow cytometric analysis of CD4+/CD25+FoxP3+ regulatory T cells. (C) Flow cytometric analysis of CD1a+/CD80+/CD86+ dendritic cells. Each bar represents the mean±s.e.m., n≥5/group.


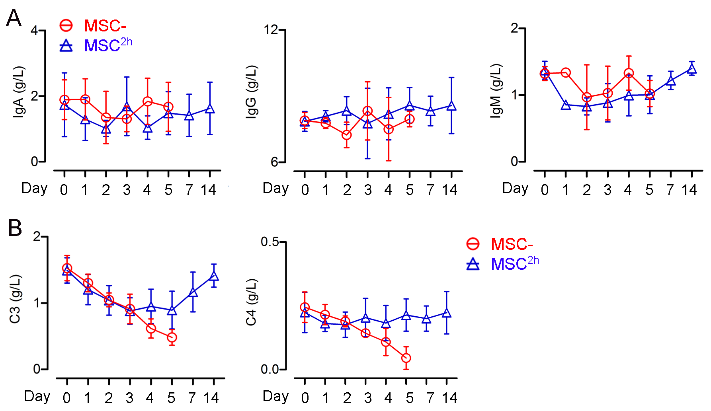


**Supplemental Figure 4.** **hUC-MSC infusion does not disturb antibodies and complements.** Student’s t test, the data are presented as the means±s.e.m., n≥5.


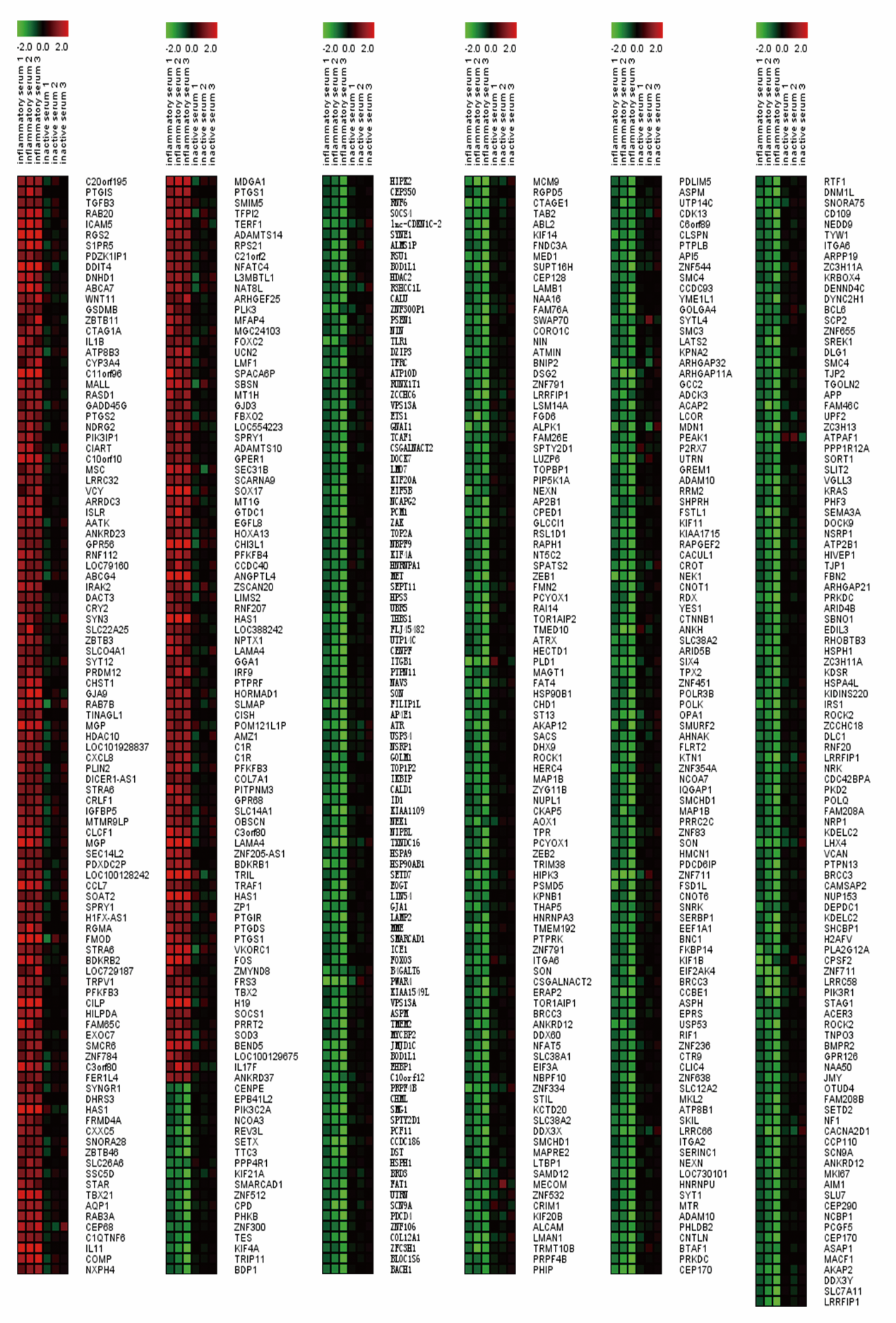
**Supporting Figure 5.** Heat map of altered genes from microarray analysis. ArrayExpress accession number: E-MTAB-4750, <http://www.ebi.ac.uk/arrayexpress/>
